# Supporting learners in prison healthcare work-integrated learning settings through simulation: a cross-sectional study

**DOI:** 10.1186/s12912-023-01506-3

**Published:** 2023-09-18

**Authors:** Judith Needham, Rhonda Beggs, Thea F. van de Mortel

**Affiliations:** 1 School of Nursing and Midwifery, Griffith University University Drive, Meadowbrook, QLD 4131 Australia; 2 School of Nursing and Midwifery, Griffith University, Kessels Rd, Nathan, QLD 4111 Australia; 3 School of Nursing and Midwifery, Griffith University, Parklands Drive, Southport, QLD 4222 Australia

**Keywords:** Prisons, Correctional facilities, Orientation, Simulation, Undergraduate student nurse, work-integrated learning

## Abstract

**Background:**

Nursing students and nurse preceptors indicate that a comprehensive orientation is vital to successful work-integrated learning placements in Prison Health Services. The aim of this study was to implement and evaluate a Prison Health Service orientation package that included innovative asynchronous online video simulations with branched decision-making and feedback opportunities to stimulate learning and improve students’ feelings of preparedness for a placement in this setting.

**Methods:**

A cross-sectional pre and post design was used to evaluate the resource. Students were given access to the package and invited to complete a pre-placement survey evaluating the resource and their feelings of preparedness for placement. Following placement, they re-evaluated the resource in terms of how well it prepared them for the placement and how well prepared they felt. Third year Australian undergraduate nursing students from one university who completed a Prison Health Service work-integrated learning placement in 2018, 2021, and 2022 were invited to participate. Placements were unavailable in 2019 and 2020. Independent t-tests were used to determine differences in scale means and level of preparedness between pre- and post-survey responses.

**Results:**

Twenty-three of 40 (57.5%) eligible nursing students completed the pre-placement survey and 13 (32.5%) completed the post placement survey. All respondents to the pre-placement survey indicated that they felt satisfactorily, well, or very well prepared after completing the orientation package prior to their clinical placement. Students were significantly more likely to consider themselves well prepared by the package after they had attended placement (p < .001). All students post placement indicated that overall, the simulation resources and the specific simulation scenario about personal boundaries and management of manipulative behaviours was useful for their placement. The majority of students would recommend the orientation package to other students. Suggestions for improvement included streamlining the resource to reduce the time to complete it.

**Conclusions:**

Asynchronous online simulation with the capacity for branched decision making and feedback along with a comprehensive online orientation package were perceived as useful to prepare undergraduate students for placement in the Prison Health Service work-integrated learning setting.

**Supplementary Information:**

The online version contains supplementary material available at 10.1186/s12912-023-01506-3.

## Background

In Australia and internationally, prisoners (those in custodial care of correctional centres) experience higher levels of mental illness, illegal drug use, chronic illnesses, communicable diseases and disability than the general community [1, 2]. Consequently, a well-skilled nursing workforce is needed to provide optimal care to this group [3]. Various studies support the suitability of work-integrated learning (WIL) placements for undergraduate nursing students in Prison Health Services (PHS) [4–6]. Work-integrated placements in non-traditional areas have been found to positively influence students’ attitude towards, and their intention to pursue careers in these areas [4, 7–10].

Evaluation of students’ experiences in PHS indicate that students are often quite anxious about the placement beforehand [5] and are at times not comfortable in the setting until their placement has nearly ended [6]. Students have requested better preparation for such placements, including more information on the possibly confronting nature of clients, the environment, and the safety and security issues inherent in the prison setting [6]. Similarly, preceptors interviewed about their experiences of supporting students in PHS, felt that students were not adequately prepared to develop therapeutic relationships in the PHS setting with clients who may have complex chronic health conditions, often come from very disadvantaged backgrounds, and who may, at times, be hostile or manipulative [11]. Both students and preceptors indicated that future students would benefit from an improved orientation to this setting. This paper reports on the outcomes of an online orientation resource to prepare students for PHS placements that incorporated branched decision-making simulation with feedback.

The wider literature also supports the need for a detailed orientation to the PHS setting, including psychological preparation and exploration of students’ feelings and expectations of the experience to improve the student experience, reducing anxiety and facilitating learning [5, 12–15]. Unique aspects of the prison (correctional) setting, including the environment, access, security and safety, institutional rules, regulations and routines, as well as law and ethics with respect to confidentiality and prisoner rights should be addressed [11, 16]. Other aspects of orientation should include safe clothing, identification, contraband, potential weapons, and interactions with prisoners, including gifts, granting requests, personal disclosure, eye contact, and maintaining professional boundaries [17].

Various authors report on the use of simulation to prepare students for or increase their capability while attending WIL in challenging settings. For example, Kunst et al. [18] found that simulation learning allowed undergraduate nursing students to develop skills for dealing with complex clinical challenges in a safe environment prior to placement in acute mental health settings; students reported improved confidence, knowledge and ability following simulation. They also found some evidence of improved student performance and reflection during placements after the simulation intervention.

Diaz et al. [17] reported that simulation using mannikins improved integration of students into the PHS setting, reducing anxiety, improving proficiency, safety and security, and enhancing nurse – patient communication by introducing students to the prison setting and allowing them to develop critical thinking about complex patients and interactions. However, their approach to simulation was in person at a set location and time - and given the distributed multi-campus nature of our program, was not suitable for our program for logistical reasons. For our cohort, online simulation might provide a mechanism that would both offer a solution to students’ need for an orientation to the PHS setting while addressing the logistical constraints of in person delivery. We could find no research into orientation of nursing students to PHS using video simulations, hence the purpose of this study was to develop, implement and evaluate an online orientation program that incorporated online simulation to determine if this would adequately prepare students for WIL in the PHS setting.

## Methods

### Intervention

To address the issue of under-preparation of our nursing students for a placement in prison health settings, an orientation package was developed by a group of experts and overseen by a governance committee. Members included PHS staff, a student representative who had previously undertaken a PHS placement, and academic staff from the Griffith University Schools of Nursing and Midwifery, and Criminology. The package was comprised of resources on a dedicated orientation website. These included video simulations using actors, and short video interviews with students and new graduates who had experienced this setting, along with a set of short online modules. Key safety aspects covered included identification, safe clothing, routine screening and safety routines, as well as potential weapons and contraband. It also addressed institutional rules and regulations, maintaining professional boundaries, ethics in relation to confidentiality and prisoner rights, and interactions with prisoners, including gifts, granting requests, personal disclosure, and eye contact. Chronic health conditions common in these settings were also addressed.

Three scripted video simulation scenarios that illustrated typical issues that might be experienced were developed by a PHS staff member, an academic staff member with a mental health background and previous experience in prison health, and a script writer from the University’s Learning Futures team. The scripts were proofread by two other academics for authenticity. The scenarios used actors to portray prisoners, correctional officers, and nursing staff, and covered a range of techniques on advanced communication, manipulation by prisoners, managing professional boundaries, and using de-escalation techniques for emotional distress. The scenarios were set in a women’s prison, a youth detention centre and a men’s prison, covering the different settings in which our students could be placed. The scenarios included decision-making trees, allowing students to work through issues and get automated feedback on their patient or situation management. The resources were accessible to students at least four weeks prior to their WIL experience. The orientation package was introduced in February 2018, and all undergraduate nursing students attending the PHS were asked to complete it prior to their clinical placement. The simulation scenarios took 25 min to complete. The videos on students’ experiences took 1.3 h to complete. There were also other helpful resources that students could access that would take 2 h to complete. We did not determine whether all students completed the package or which components they completed.

### Design and participants

A cross-sectional design was adopted. All 40 nursing students who were scheduled to complete a prison placement between February and December in 2018, 2021 and 2022 were invited via email to participate the study. Students were asked to complete an anonymous pre-placement survey (Supplementary file 1) after completing the orientation package and before attending placement. Following placement, they were asked to complete an anonymous post-placement survey (Supplementary file 2). The surveys directly addressed their perceptions of the usefulness of the simulated learning package as an orientation tool. No data were collected in 2019–2020 as PHS placements were not available in those years.

### Data collection and survey instrument

A link to each survey was emailed to students along with an information sheet that explained the survey purpose. Survey questions incorporated fixed-response questions using 5-point Likert scales, and provision was also made for students to make open-ended responses to all sections of the surveys. The 5-point Likert scales ranged from strongly disagree to strongly agree for questions related to individual elements of the orientation package, and totally unprepared, poorly prepared, satisfactorily prepared, well prepared, and very well prepared for their evaluation of overall preparedness for the PHS placement. Two reminder emails were sent to students to increase the response rate.

### Setting

Students were placed in a metropolitan PHS, at one of three maximum-security sites: a women’s prison, and two men’s prisons. Sites accepted between one and four students per placement. Registered nurses precepted the students.

### Ethical considerations

Ethics approval to conduct the study was obtained from the Griffith University Human Research Ethics Committee (Ref No: GU2017/280; Ref No: GU2022/141). Students were provided with an information sheet that explained the study purpose and what was involved in the study. Participation was voluntary, the survey was anonymous and online, and survey completion was considered to indicate consent.

### Data analysis

Descriptive statistics were calculated for quantitative data (mean and standard deviation). Independent samples t-tests were used to compare means between pre and post WIL samples. Cronbach’s alpha was calculated for scales to determine scale reliability. Content analysis was used to examine the qualitative data obtained from student responses to open-ended questions. Two researchers independently extracted meaning units from the transcripts related to the study aims. Meaning units were grouped into themes [19]. This independent review contributed to data analysis trustworthiness. Themes were then discussed between the researchers contributing to the research credibility.

## Results

### Pre-placement survey

Twenty-three of 40 eligible students responded to the pre-placement survey (57.5% response rate). All were female; 12 (52.2%) were aged under 30, four (17.4%) 30–39, and seven (30.4%) were aged 40 or more. Pre-placement scale reliability measured via Cronbach’s alpha ranged from 0.97 to 0.99. Participants were asked to indicate their level of agreement with statements about the appropriateness and utility of the simulation resources (Table 1) and the content and usefulness of orientation resources (Table 2) prior to their placement. The students were largely positive about the simulation resources (mean 4.2/5 ± 0.8). Twenty-two (95.7%) agreed or strongly agreed that the simulation resources were appropriate to prepare them for a PHS placement, and that the content helped them think about situations they might encounter; 21 (87%) found it useful to hear about experiences of students who had already attended a PHS placement and would recommend the simulation resources to other students.

**Table 1:** Pre-placement agreement with statements about simulation resources

Item	Mean (SD)	
The content was appropriate for me as a student nurse planning for my prison health service clinical placement	4.1 (0.8)	
The case scenarios were realistic	4.1 (0.9)	
The scenario about Risk Assessment and Management of Emotional Distress was useful for my clinical placement preparation	4.0 (1.0)	
The scenario about Personal Boundaries and Management of Manipulative Behaviours was useful for my clinical placement preparation	4.3 (0.9)	
The scenario about De-escalation and Anger Management was useful for my clinical placement preparation	4.3 (0.9)	
It was useful to hear about the experiences of students who had already attended a clinical placement in a prison health service	4.5 (0.9)	
The content helped me think about what situations I might encounter when on clinical placement	4.4 (0.9)	
The content helped prepare me psychologically for my prison health service clinical placement	4.1 (1.1)	
I would recommend these simulation resources to other students	4.4 (09)	
TOTAL	4.2 (0.8)	

Nursing students were largely positive about the orientation resources (mean 4.2/5 ± 0.8); 21 (87%) felt the content was appropriate and that they would recommend the orientation package to other students. In response to the question regarding whether, all things considered, participants felt prepared for their PHS clinical placement, none felt totally unprepared or poorly prepared, 10 (45.5%) felt satisfactorily prepared, 10 (45.5%) felt well prepared, and three (9.1%) very well prepared (mean 3.6/5 ± 0.7).

**Table 2:** Pre-placement agreement with statements about the content and usefulness of orientation resources

Item	Mean (S.D)
The content was appropriate	4.2 (0.9)
The Introduction section was useful	4.3 (0.9)
The Preparation section was useful	4.1 (0.9)
The Safety section was useful	4.2 (0.9)
The Communication section was useful	4.1 (0. 9)
The Activities section was useful	4.2 (0. 9)
The References section was useful	4.1 (0. 9)
The orientation resources covered everything I think should be covered	4.0 (0.9)
I would recommend these orientation resources to other students	4.2 (0.9)
TOTAL	4.2 (0.8)

Analysis of open-ended comments related to the simulation and orientation resources produced two themes: the resources were useful and appreciated, and improvements could be made. Examples of positive comments included;

*Videos from students were good* (S10),

*Any help is great, it is unknown territory for student… It is very useful preparation* [for] *prison placements (S18)*.

*“Really useful…really helpful to prepare going there (S14)“*.

One student stated that they *“found [the scenarios] useful…probably a little too long”* (S4). *Others suggested adding*:

“...*quizzes to test [students’] knowledge”* (S10)

“*...more information about the first/orientation day, and the shift hours in correctional centre for nursing staff”* (S14).

“*more on manipulative behaviours (e.g., what to look out for”* (S15)

“*a simulation that is a rundown of the correctional nurse’s day. What duties the nurse needs to complete during their shift*” (S11).

*“exactly what you can and can’t take…it states you cannot take a mobile phone into the prison itself but does not state if this must be left at home altogether or if there is storage for items”* (S7).

### Post-placement survey

Thirteen of 40 students responded to the post-placement survey (32.5% response rate). Nine were aged under 30, one was aged 30–39, two were aged 40 or above, and one did not indicate their age. Ten were female, two were male, and one did not answer. Twelve respondents were placed in men’s prisons, and one in a women’s prison.

Participants were asked to indicate their level of agreement with statements about the simulation orientation resources (Table 3) and the orientation website (Table 4) following WIL. All 13 (100%) agreed or strongly agreed that overall, the simulation resources provided useful preparation for their PHS placement, and that the simulation scenario about personal boundaries and management of manipulative behaviours was useful for their placement (Table 3). Ten (76.9%) agreed that they would recommend the simulation resources to other students while three were unsure. The simulation scenarios were highly rated by students. Fewer students agreed that the simulation resources helped to prepare them psychologically for the placement (3.7/5 ± 1.0) or deal with specific situations (3.5/5 ± 1.3), although the majority agreed with those statements.

**Table 3:** Post-placement level of agreement with statements about simulation resources

Item	Mean (S.D.)
The content was appropriate for a student nurse attending a prison health service clinical placement	4.1 (1.2)
The case scenarios were realistic	4.1 (0.8)
The Risk Assessment & Management of Emotional Distress scenario was useful for my clinical placement	4.1 (0.5)
The Personal Boundaries and Management of Manipulative Behaviours scenario was useful for my clinical placement	4.4 (0.5)
The De-escalation and Anger Management scenario was useful for my clinical placement	4.1 (0.8)
It was useful to hear about the experiences of students who had already attended a placement in a prison health service	4.3 (0.8)
The content helped me deal with situations I encountered when on clinical placement	3.5 (1.3)
The content helped prepare me psychologically for my prison health service clinical placement	3.7 (1.0)
I would recommend these simulation resources to other students	4.2 (0.8)
Overall, the simulation resources were useful preparation for my placement	4.5 (0.5)
TOTAL	4.0 (0.7)

In terms of the utility of the simulation, one student indicated, “*it seemed a bit unrealistic and stereotyped*” (S8), another indicated “*there is a wide variation between the way things were dealt with in the orientation resources and the way they are actually dealt with in the prison*” (S5), while another stated, “*I felt I was well prepared to come onto the placement*” (S2).

In terms of the orientation resources, students agreed that the various sections of the orientation website were useful (mean 4.2 ± 0.5) and 12/13 (92.3%) indicated they would recommend the orientation website to other students (Table 4).

**Table 4:** Post-placement level of agreement with statements about the content and usefulness of orientation resources

Item	Mean (S.D.)
The Introduction section was useful	4.2 (0.4)
The Preparation section was useful	4.2 (0.4)
The Safety section was useful	4.2 (0.4)
The Communication section was useful	4.2 (0.6)
The Activities section was useful	4.0 (0.4)
The orientation resources covered everything I think should be covered	4.2 (0.6)
I would recommend the orientation website to other students	4.2 (0.6)
TOTAL	4.2 (0.5)

In response to the questions on potential improvements, students suggested streamlining the resource and/or making it more accessible:

‘*To be …shorter, due to the already high load of work [in] third year…second semester”* (S1).

*“They could flow from one to another better by not having to click in and out of sections”* (S2).

“*There is a lot of information… I think… printed material…should be used because it gives the student time to read over the material again before they start placement*” (S3).

Another suggested including, *“a competency quiz to be completed before starting placement” (S2).*

All post-placement participants rated their preparation for the PHS clinical placement positively; seven (53.8%) agreed/strongly agreed that they were well prepared and six (46.2%) agreed/strongly agreed that they were very well prepared (mean 4.46/5 ± 0.52). While simulation and orientation scale means did not differ significantly pre and post placement, participants post-placement were significantly more likely to agree/strongly agree that they were well prepared for a placement in PHS than they were pre-placement (95% CI -1.3 - -0.4; mean difference − 0.9; p < .001). Post-placement scale reliability ranged from 0.90 − 0.96.

## Discussion

Nursing students in this study supported the use of the online orientation package to the PHS setting, which included simulations using a branched-outcome decision model, allowing students to choose response options and receive automated feedback on their choices. This approach has previously been found to be successful for pharmacy students in other healthcare settings, with improvements seen in their knowledge and skills, critical thinking and analytical skills [20, 21], and with nursing students, improving their self-confidence in their learning when employed in the classroom setting [22].

Scores on the scales did not differ significantly from pre to post surveys. The students largely perceived the resources to be helpful prior to attending placement and perceived them to be equally helpful after attending placement. However, their feelings of preparedness increased significantly. Of the students who completed the pre-placement survey following completion of the orientation package, 60% agreed they were satisfactorily prepared, 30% felt well prepared, and 10% felt very well prepared for their placement. Post-placement all participants in this study indicated that they felt well, or very well prepared for their PHS placement and agreed or strongly agreed that the simulation resources were useful preparation overall. Most felt the orientation resources covered everything they thought should be covered and would recommend the orientation website to other students. A couple of students indicated they felt the simulation scenarios were unrealistic in their qualitative comments. The scenarios - which were constructed based on the experiences of three PHS nurse educators and examples from the literature - were designed to cover some specific behaviours and techniques, i.e., manipulation of staff by prisoners, managing emotional distress, and de-escalation techniques. Some students, in the comparatively short time they were on placement, may not have necessarily encountered these behaviours (and therefore have considered them unrealistic), but the aim was to prepare them in case they did encounter them.

McCloughen et al. [23] indicate that regulation of emotions on placement is stressful for student nurses. Given our students gave a lower rating to feeling psychologically prepared for the PHS placement (3.7/5), more work is needed to understand and address the gaps in the resource to prepare students for these emotions and provide strategies for dealing with them.

A Western Australian study [24] found that over half the undergraduate nursing students surveyed experienced non-physical and physical aggression during WIL placements and concluded that nursing curricula need to better prepare student nurses to confront such experiences and build resilience to aggression and violence. Online courses have proved effective in educating student nurses about workplace violence in other health care settings [25]. The videos using actors to illustrate techniques for de-escalation, managing difficult behaviours, and maintaining boundaries may be useful in the broader undergraduate general nursing setting, as the techniques used are similar regardless of the group and setting in which they are applied.

Ashton [26] recommends that all student nurses be educated about the challenges specific to caring for prisoners, as many will encounter them in the acute care setting. However, those preparing for a PHS WIL placement require a more extensive orientation process to address the issues inherent in the PHS environment. Previous research [6] demonstrates that while students placed in PHS WIL placements have had largely positive experiences, they have struggled with aspects of the correctional environment, taking longer to integrate into the workplace than in less challenging environments, and having difficulties developing the therapeutic relationship with prisoner patients. Additionally, new registered nurse recruits to PHS [27–30] and nurses transitioning from other areas [31] also strongly benefit from orientation addressing challenges specific to the environment, specific organisational knowledge, role expectations, workplace familiarisation and new technology. Limited nursing orientation has been associated with poor retention of registered nurses in PHS [27, 29, 30].

The simulations were available online, making the orientation package more sustainable than using mannikins, which Diaz et al. [17] found successful but which required a physical staff presence to engage students with the simulation. Mannikin simulations have also been found to be costly [32]. Our approach made the learning package cheaper, more accessible, and able to be completed anywhere and at the student’s convenience. In response to students’ suggestions, we will add an option to download module content.

Given that positive experiences during WIL placements are directly related to student nurses’ future intentions to work in challenging areas [7, 10, 33], providing a thorough orientation program may help future recruitment in PHS.

### Limitations

This small study conducted with nursing students from one university in an Australian metropolitan PHS may not be generalised to other prisons and student cohorts. Given students were not identified to preserve their anonymity, it is likely that some different students completed the pre-and post-placement surveys, and it’s possible that there was selection bias.

## Conclusions

Work-integrated learning placements in PHS provide rich learning opportunities for nursing students. However, nursing students and learning facilitators have indicated a robust orientation to this non-traditional placement setting is required for successful learning. While simulation is widely used in health education, this project is innovative as it involves asynchronous online simulation with the capacity for branched decision making and feedback in a setting where it has not previously been used, i.e. preparing students for placement in the non-traditional PHS clinical placement.

This model of student preparation for WIL may be transferable to other settings. The videos illustrating techniques for de-escalation, managing difficult behaviours, and maintaining boundaries may be useful outside the undergraduate nursing PHS WIL placement setting, as the techniques used are similar regardless of the group and setting in which they are applied.

### Electronic supplementary material

Below is the link to the electronic supplementary material.

Supplementary Material 1

Supplementary Material 2

## Data Availability

The dataset used during the study are available from the corresponding author on reasonable request.
