# Supplementary material for: Supporting learners in prison healthcare work-integrated learning settings through simulation: a cross-sectional study
Source: BMC Nurs. 2023 Sep 18;22:322. doi: 10.1186/s12912-023-01506-3 (PMC10506296; doi:10.1186/s12912-023-01506-3)
Supplement: Supplementary file 1 — Supplementary Material 1 [file 12912_2023_1506_MOESM1_ESM.pdf]

Griffith UNIVERSITY

Copy of An evaluation of orientation activities to prepare students for clinical placements in Prison Health Services

1. Please indicate your level of agreement with the following statements about the simulation resources.

	Strongly disagree	Disagree	Not sure	Agree	Strongly agree
The content was appropriate for me as a student nurse planning for my prison health service clinical placement	○	○	○	○	○
The case scenarios were realistic	○	○	○	○	○
The scenario about Risk Assessment and Management of Emotional Distress was useful for my clinical placement preparation	○	○	○	○	○
The scenario about Personal Boundaries and Management of Manipulative Behaviours was useful for my clinical placement preparation	○	○	○	○	○
The scenario about De-escalation and Anger Management was useful for my clinical placement preparation	○	○	○	○	○
It was useful to hear about the experiences of students who had already attended a clinical placement at a prison health service	○	○	○	○	○
The content helped me think about what situations I might encounter when on clinical placement	○	○	○	○	○
The content helped prepare me psychologically for my prison health services clinical placement	○	○	○	○	○
I would recommend these simulation resources to other students	○	○	○	○	○

2. What, if any, other comments do you have about the content and usefulness of the simulation resources?

3. What areas of improvement, if any, can you suggest for the simulation resources?

4. Please indicate your level of agreement with the following statements about the orientation resources

	Strongly disagree	Disagree	Not sure	Agree	Strongly Agree
The content was appropriate	○	○	○	○	○
The Introduction section was useful	○	○	○	○	○
The Preparation section was useful	○	○	○	○	○
The Safety section was useful	○	○	○	○	○
The Communication section was useful	○	○	○	○	○
The Activities section was useful	○	○	○	○	○
The References section was useful	○	○	○	○	○
The orientation resources covered everything I think should be covered	○	○	○	○	○
I would recommend these orientation resources to other students	○	○	○	○	○

5. What, if any, other comments do you have about the content and usefulness of the orientation resources?

6. What areas of improvement, if any, can you suggest for the orientation resources?

7. All things considered, how well prepared do you feel for your Prison Health Service clinical placement?

- ◯ Totally unprepared
- ◯ Poorly prepared
- ◯ Satisfactorily prepared
- ◯ Well prepared
- ◯ Very well prepared

8. Please share any other comments about your clinical placement preparation here.

[ ]

9. Your gender

- ◯ Male
- ◯ Female

10. Your age

- ◯ < 20
- ◯ 20-29
- ◯ 30-39
- ◯ 40-49
- ◯ 50 or more

11. What year did you commence your Bachelor of Nursing?

[ ]

12. Which course is your Prison Health Service clinical placement associated with?

[ ]

Thank you very much for your feedback and participation in this evaluation. Improving our program is an important part of increasing the value of your educational experience and your degree. We appreciate your involvement!
