# Supplementary material for: Supporting learners in prison healthcare work-integrated learning settings through simulation: a cross-sectional study
Source: BMC Nurs. 2023 Sep 18;22:322. doi: 10.1186/s12912-023-01506-3 (PMC10506296; doi:10.1186/s12912-023-01506-3)
Supplement: Supplementary file 2 — Supplementary Material 2 [file 12912_2023_1506_MOESM2_ESM.pdf]

# An evaluation of preparedness for clinical placements in Prison Health Services

## Appropriateness of placement

**The following section pertains to your experience within the health centre where you did your clinical placement and how well prepared you felt.**

1. Please identify the name of the health centre where you did your clinical placement.

Questions evaluating the placement  iteself have been removed.

8. Now that you have attended placement, please indicate your level of agreement with the following statements about the **simulation** orientation resources (i.e. the videos).

| | Strongly disagree | Disagree | Not sure | Agree | Strongly agree |
|---|---|---|---|---|---|
| The content was appropriate for a student nurse attending a prison health service clinical placement | ○ | ○ | ○ | ○ | ○ |
| The case scenarios were realistic | ○ | ○ | ○ | ○ | ○ |
| The scenario about | | | | | |

| | | | | | |
|---|---|---|---|---|---|
| Risk Assessment and Management of Emotional Distress was useful preparation for my clinical placement | ○ | ○ | ○ | ○ | ○ |
| The scenario about Personal Boundaries and Management of Manipulative Behaviours was useful preparation for my clinical placement | ○ | ○ | ○ | ○ | ○ |
| The scenario about De-escalation and Anger Management was useful preparation for my clinical placement | ○ | ○ | ○ | ○ | ○ |
| It was useful to hear about the experiences of students who had already attended a clinical placement at a prison health service | ○ | ○ | ○ | ○ | ○ |
| The content helped me deal with situations I encountered on clinical placement | ○ | ○ | ○ | ○ | ○ |
| The content helped prepare me psychologically for my prison health clinical placement | ○ | ○ | ○ | ○ | ○ |
| I would recommend these simulation resources to other students | ○ | ○ | ○ | ○ | ○ |
| Overall the simulation resources were useful preparation for my placement | ○ | ○ | ○ | ○ | ○ |

9. Please indicate your level of agreement with the following statements about the **orientation website**

| | Strongly disagree | Disagree | Not sure | Agree | Strongly Agree |
|---|---|---|---|---|---|
| The Introduction section was useful | ○ | ○ | ○ | ○ | ○ |
| The Preparation section was useful | ○ | ○ | ○ | ○ | ○ |
| The Safety section was useful | ○ | ○ | ○ | ○ | ○ |
| The Communication section was useful | ○ | ○ | ○ | ○ | ○ |
| The Activities section was useful | ○ | ○ | ○ | ○ | ○ |
| The orientation resources covered everything I think should be covered | ○ | ○ | ○ | ○ | ○ |
| I would recommend the orientation website to other students | ○ | ○ | ○ | ○ | ○ |

10. What areas of improvement, if any, can you suggest for the simulation videos and orientation website?

11. What, if any, other comments do you have about the content and usefulness of the simulation and orientation resources?

12. All things considered, how well prepared were you for your Prison Health Service clinical placement?

- ○ Totally unprepared
- ○ Poorly prepared
- ○ Satisfactorily prepared
- ○ Well prepared
- ○ Very well prepared

13. Please share any other comments about your clinical placement preparation here.

14. Your age

- ◯ < 20
- ◯ 20-29
- ◯ 30-39
- ◯ 40-49
- ◯ 50 or more

15. Your gender

- ◯ Female
- ◯ Male
- ◯ Other

Thank you very much for your feedback and participation in this evaluation. Improving our program is an important part of increasing the value of your educational experience and your degree. We appreciate your involvement!
